# A Reference Handbook of the Medical Sciences

**Published:** 1903-12

**Authors:** 


A REFERENCE HANDBOOK OF THE MEDICAL SCIENCES. Embracing the entire range of scientific and practical medicine and allied science. By various writers. A new edition, completely revised and rewritten. Edited by Albert H. Buck, M. D., New York city. Volume VI, illustrated by chromolithographs and seven hundred and sixty-three half-tone engravings. Price, per volume, ———. New York: William Wood & Co. 1903.

This volume contains 1004 pages, being somewhat larger than the preceding volumes, but in every way equal to them.

It is hardly possible to select for separate mention any particular articles, the contents of the book is of so high a standard. One might, however, mention the articles on "Muscles," by Prof. Simon H. Gage, Lydia M. Dewitt and Francis J. Shephard; on "Nasal Cavities," by Robert C. Myles; on "Parasites," by Henry B. Ward; on the "Nervous System," by Pearce Bailey, James J. Putnam, Wm. M. Leszynsky and Lewellys F. Barker; on "Opthalmological Topics," by John Green and Beaumont Small; on "Ovariotomy," by Hunter Robb; on the "Ovum," by Robert P. Bigelow; on the "Pelvis," by Frank Baker; on the "Pharynx," by Seth Scott Bishop; on "Pleurisy," by Frank W. Jackson; on "Pneumonia," by Andrew H. Smith; on "Poisonous Plants," by Henry H. Bushby; on "Poisonous Reptiles," by Gustav Langmann; on "Poisons," by Charles Harrington and R. H. C. Chittenden; on the "Prostate," by Arthur T. and Hugh Cabot; on "Ptomains," by Rudolph A. Witthaus; on the "Pulse," by Wm. S. Morrow; on "Reflexes," by Joseph Fraenkel; on "Reparative Surgery," by Joseph Ransohoff; on "Resection of Joints," by Frank Hartley, and on "Roentgen Rays," by Wm. C. Borden. This volume contains 764 engravings and nine insert plates in black and colors.

